# Inflammasome: A Double-Edged Sword in Liver Diseases

**DOI:** 10.3389/fimmu.2018.02201

**Published:** 2018-09-25

**Authors:** Jingyun Luan, Dianwen Ju

**Affiliations:** Department of Microbiological and Biochemical Pharmacy & The Key Laboratory of Smart Drug Delivery, Ministry of Education, School of Pharmacy, Fudan University, Shanghai, China

**Keywords:** inflammasome, liver, pyroptosis, innate immune, adaptive immune, metabolism

## Abstract

Inflammasomes have emerged as critical innate sensors of host immune that defense against pathogen infection, metabolism syndrome, cellular stress and cancer metastasis in the liver. The assembly of inflammasome activates caspase-1, which promotes the maturation of interleukin-1β (IL-1β) and interleukin-18 (IL-18), and initiates pyroptotic cell death (pyroptosis). IL-18 exerts pleiotropic effects on hepatic NK cells, priming FasL-mediated cytotoxicity, and interferon-γ (IFN-γ)-dependent responses to prevent the development of liver diseases. However, considerable attention has been attracted to the pathogenic role of inflammasomes in various acute and chronic liver diseases, including viral hepatitis, nanoparticle-induced liver injury, alcoholic and non-alcoholic steatohepatitis. In this review, we summarize the latest advances on the physiological and pathological roles of inflammasomes for further development of inflammasome-based therapeutic strategies for human liver diseases.

## Introduction

Innate immune system is well-known as the first line defense against pathogen associated molecular patterns (PAMPs) derived from microbial pathogens (e.g., bacteria, parasites, viruses) and damage associated molecular patterns (DAMPs) produced by host cells (e.g., cellular stress, cytosolic DNA, damage) (1, 2). Inflammasomes are critical innate immune sensors involved in maintaining the cellular health in response to cytosolic pathogens or stress signals (3). Inflammasomes are cytoplasmic multiprotein complexes typically composed of three components: (i) a sensor molecule consisting of NOD-like receptors (NLRs), absent in melanoma 2 (AIM2) or pyrin, (ii) an adaptor protein, and (iii) an effector molecule procaspase-1 (4). Upon stimulation, inflammasome complexes are assembled to process the cleavage of caspase-1, which activates proinflammatory cytokines interleukin-1β (IL-1β) and interleukin-18 (IL-18), and a cytosolic protein gasdermin D (GSDMD) (5). Cleaved GSDMD forms pores on the plasma membrane, which induces pyroptotic cell death and permits the release of IL-1β and IL-18 into the extracellular space (6, 7). These two proinflammatory cytokines and pyroptosis exert both beneficial and deleterious effects in the liver, which will be discussed in this review.

The liver is a “first past” organ that continually challenged with diverse microbial particles derived from intestine through the portal circulation. During this process, a large number of cytosolic pathogens can be sensed by inflammasomes, which are pivotal in evoking adaptive immunity for complete clearance (8–10). In addition, hepatocytes are susceptible to the infection of various liver-tropic viruses that leads to the pathogenesis of virus-related liver diseases. The emerging importance of inflammasomes in response to viral infection points to another area for the involvement of inflammasomes (11).

Paradoxically, inflammasomes are essential for liver defense against pathogens and danger signals, but excessive activation of inflammasomes promotes the pathogenesis of various liver diseases (Table 1). Thus, a definitive understanding of the roles of inflammasomes in the liver is essential for the development of inflammasome-based therapies.

**Table 1:** Roles of inflammasomes in the liver diseases.

**Inflammable**	**Physiological roles in liver diseases**	**Pathological roles in liver diseases**	**References**
NLRP1	Prevent obesity and metabolic syndrome	Remain unclear	(12, 13)
NLRP3	Prime NK cell tumoricidal activity to inhibit liver CRC metastatic growth; prime NK cell IFN-γ production to suppress HCV replication; module intestinal microbiota to suppress the progression of NAFLD; trigger pyroptosis of HCC cells	Induce pyroptosis and activate iNKT cells to promote liver injury during ALD; amplify inflammatory responses and induce liver fibrogenesis in NASH; activate lipogenesis to promote replication of HCV; activate HSCs to promote liver fibrosis; induce pyroptosis and trigger hepatocytes injury in response to nanoparticles	(14–17) (18–26)
NLRC4	Prime NK cells cytotoxicity to counteract the infection of *C. violaceum;* promote liver regeneration after partial hepatectomy and decrease liver fibrosis	Promote the development of ALD	(27, 28) (29)
NLRP6	Modulate intestinal microbiota to suppress the progression of NAFLD	Suppress NF-κB and MAP-kinase activation and increase bacterial burdens in the liver in response to *Listeria monocytogenes* and *Salmonella typhimurium*	(15) (30)
NLRP12	Attenuate inflammation responses during hepatic ischemia/reperfusion injury	Suppress phosphorylation of IκBα and ERK and increase susceptibility to *Salmonella*	(31) (32)
AIM2	Activate caspase-1 to protect hepatocytes from redox stress-induced injury; inhibit the activation of EMT to suppress HCC metastasis	Exacerbate inflammation in macrophages isolated from ascitic fluid of patients with cirrhosis	(33, 34) (35)

*CRC, colorectal cancer; HCC, hepatocellular carcinoma; IFN-γ, interferon-γ; HCV, hepatitis C virus; NAFLD, non-alcoholic fatty liver disease; NASH, nonalcoholic steatohepatitis; ALD, alcoholic liver disease; HSCs, hepatic stellate cells; C. violaceum, Chromobacterium violaceum; EMT, epithelial-mesenchymal transition*.

## Physiological roles of inflammasomes in the liver

### Inflammasome-mediated liver defense against bacterial and parasitic infections

Continual exposure to orally-ingested antigens and intestinally-released microbial products sensitizes the liver to various bacterial and parasitic infections. Inflammasomes assembled by NLRs are known as innate immune sensors that detect cytosolic contaminations or perturbations (36, 37). NLRC4 is demonstrated to defense against bacterial infections through detecting bacterial flagellin or two bacterial type III secretion systems (T3SSs) (38–42). Studies on the liver-tropic pathogen *Chromobacterium violaceum* have shown that NLRC4-deficient mice are more susceptible to infection (27). Activated NLRC4 inflammasome processes the production of IL-18, which primes NK cell cytotoxicity to clear hepatocyte replication niches (27). Additionally, exogenous administration of IL-18 is protective against the infection of *C. violaceum* and another liver-tropic bacteria *Listeria monocytogenes* (27). Although IL-18 therapy does not fully reduce bacterial burdens to the normal level, considerable potential still exists in activating NLRC4-IL-18 pathway as a combination therapy to counteract bacteria with T3SSs or flagellin.

In addition to participating in liver defense against bacterial pathogens, inflammasomes are also identified to control parasitic infections. Canonical inflammasomes process the cleavage of caspase-1, while noncanonical inflammasomes are implicated in the activation of caspase-11 (43, 44). Loss of all inflammasome signalings by knocking out caspase-1/11 leads to higher hepatic parasitic load and lower survival of mice in response to *Trypanosoma cruzi* (*T. cruzi*) (45, 46). The activation of caspase-1 is highly dependent on lysosomal cathepsin B, as pharmacological inhibition of cathepsin B significantly reduces the production of IL-1β during *T. cruzi* infection (45). Furthermore, the lack of IL-1β in caspase-1/11-deficient mice is accompanied by downregulated hepatic interleukin-17+CD8+ and interferon-γ (IFN-γ)+CD8+ T cells, implying that inflammasome-mediated IL-1β is involved in promoting liver adaptive immunity to control this parasitic infection (46). This finding is in line with previous studies showing the importance of IL-1β for the differentiation of Th17 cells and antigen-driven T cells (47–49). Thus, the interplay between inflammasomes and adaptive immune system in the defense against pathogen infection is an interesting aspect for future investigations.

### Inflammasome-mediated inhibition of hepatitis virus infection

Viral infection typically initiates a cascade of innate immune responses, which restrict viral spread and provoke adaptive immunity for complete removal of virus. As innate immune sensors, inflammasomes have a prominent role in defense against hepatitis virus infections (14, 50, 51). Mice deficient in caspase-1/11 are more susceptible to mouse hepatitis virus (MHV) infection, suggesting that inflammasomes as a whole are protective (51). Further explorations have identified that inflammasome-dependent cytokine, IL-18, is required for host defense against MHV, as IL-18 receptor (IL-18R) deficiency reduces the production of IFN-γ by activated T cells, causing elevated viral replication and poor survival of MHV-infected mice (51). Despite evidence for the involvement of IL-18 in MHV inhibition, many unanswered questions remain, including which inflammasome mediates the maturation of IL-18 and how this inflammasome is activated during MHV infection.

Beyond T cells, IL-18 is also involved in promoting the production of IFN-γ by NK cells (14). Studies performed on an *in vitro* model of hepatitis C virus (HCV) replication have shown that monocytes can detect HCV-infected hepatocytes and respond by secreting IL-18 in an NLRP3 inflammasome-dependent manner, which subsequently stimulates NK cell-derived IFN-γ, causing suppression on HCV (14). This finding is supported by the observation of a higher expression of IL-18R on NK cells than other cell populations (27). However, this protective effect seems to be specific for monocytes, because macrophage-derived IL-1β amplifies inflammatory responses during HCV infection (52). Thus, special consideration is required for inflammasome-related inflammatory responses when applying inflammasome-based therapies for hepatitis virus infection.

### Inflammasome-mediated hepatocellular protection against oxidative stress

Oxidative stress induced by excessive reactive oxygen species (ROS) has emerged as a hallmark of liver injury (53). During this process, nuclear DNA and mitochondrial DNA (mtDNA) are released into the cytosol which act as DAMPs to activate innate immunity (54). Recent progress has been made in identifying that inflammasomes have a broader role in protecting hepatocytes from oxidative stress-induced injury by responding to mtDNA (55, 56). For example, activation of caspase-1 increases resistance to oxidative stress-induced liver inflammation and damage during hemorrhagic shock with resuscitation (HS/R) (55). Follow-up studies have shown that activation of caspase-1 is mediated by AIM2 inflammasome, as AIM2 deficiency reduces the production of caspase-1 and aggravates hepatocellular cell death (33). Considering that AIM2 inflammasome is an intracellular receptor that recognizes dysfunctional DNA, further investigations have demonstrated that AIM2 interacts with the immunogenic DNA sensor, high mobility group box 1 (HMGB1), to facilitate hepatoprotective effects (33). Another interesting finding is that AIM2 inflammasome-mediated caspase-1 upregulates the expression of beclin-1, which initiates autophagy to clear damaged mitochondria in hepatocytes, thus reducing the generation of ROS and degrading damaged mtDNA (33, 55). Together, these findings suggest that AIM2 inflammasome is on the crossroad of innate immune sensor and beneficial autophagy to protect against oxidative stress-induced liver injury. In addition, the emerging importance of hepatocytes (nonimmune cell type) in regulation of the immune response is another important area for further investigations.

### Inflammasome-mediated prevention against hepatic lipid metabolism

One additional organ-specific feature of liver that makes it sensitive to inflammasome is that liver serves as the second largest storehouse of lipid next to adipose tissues (57). Emerging evidences have indicated that inflammasomes are critical regulators in suppressing hepatic lipid deposition (58, 59). Mice deficient in NLRP1 spontaneously develop hepatic steatosis, and the syndrome is aggravated on high-fat diet (HFD), while *NLRP1*^*MUT*^ (an activating mutation in NLRP1a) mice are devoid of lipid vacuoles in the liver (12). The anti-obesity ability of NLRP1 inflammasome appears to be dependent on IL-18, as knockout of IL-18 reverses its protective effects (12, 13). In addition, exogenous administration of IL-18 counteracts steatohepatitis in mice upon HFD, further highlighting the importance of NLRP1-IL-18 signaling in controlling metabolic syndromes (12). From the mechanistic perspective, these studies also raise an interesting question about what is the trigger that leads to the activation of NLRP1 inflammasome in metabolic liver diseases, which needs to be more clearly elucidated.

Another two inflammasomes, NLRP3 and NLRP6, have also been reported to negatively regulate the progression of non-alcoholic fatty liver disease (NAFLD) (15). NLRP3- or NLRP6-deficient mice develop exacerbated hepatic steatosis with a microbiome dysbiosis on either HFD or methionine-choline-deficient diet (MCDD) (15). Interestingly, the microbiome dysbiosis in *NLRP3*^−/−^ or *NLRP6*^−/−^ mice can be transferred to co-housed wide type (WT) mice and is strongly correlated with the severity of hepatic disorders (15). A possible explanation is that exposure to HFD or MCDD alters the gut microbiota composition and function, which increase the translocation of bacterial products into the liver, thus aggravating hepatic steatosis and inflammation, termed “gut-liver axis” (60–62). NLRP6 inflammasome is recognized as a potent modulator for maintaining gut homeostasis (63). One group of microbial-derived metabolites triggers the activation of NLRP6-caspase-1 axis and subsequently leads to the proteolytic processing of IL-18 (63). IL-18 not only elicits anti-microbial peptides synthesis to control the composition of the gut microbiota, but also upregulates interleukin-22 signaling to promote wound healing (63, 64). The uncovered interactions between inflammasomes and hepatic lipid metabolism provide novel therapeutic targets for the treatment of hepatic steatosis.

### Inflammasome-mediated suppression of hepatic tumor growth

NLRP3 inflammasome has been recognized to be important for tumor control by directly activating pyroptotic cell death or secreting death-inducing cytokines (65). Evidence of NLRP3 inflammasome in suppressing hepatic tumor growth comes from the study showing that colorectal cancer (CRC) metastatic liver tumor burden is exacerbated in NLRP3-deficient mice (16). The tumor-suppressive effect of NLRP3 inflammasome on liver CRC metastasis is highly dependent on IL-18, which promotes the maturation of hepatic NK cells and primes FasL-mediated cytotoxicity (16, 17). This study provides insight into the innate immunity circulation between CRC-induced activation of NLRP3 inflammasome in kupffer cell- and NK cell-mediated cytotoxicity. In addition to priming tumoricidal activity of hepatic NK cell, NLRP3 inflammasome also induces caspase-1-mediated pyroptosis to control the proliferation of hepatocellular carcinoma (HCC) cells (66). Downregulated expression of NLRP3 inflammasome in HCC tissues correlates with the aggravation of carcinoma, while reconstitution of NLRP3 inflammasome dramatically reverses the malignant phenotype of HCC (67). These findings highlight the significance of NLRP3 inflammasome in preventing hepatic tumor growth, but it also exerts pro-carcinogenic effects for gastric and prostate cancers, indicting the protective role of NLRP3 inflammasome in cancer development may be organ or cell specific (65).

## Pathological roles of inflammasomes in hepatic diseases

### Alcoholic liver disease (ALD)

ALD is triggered by excessive alcohol consumption that can progress from fatty liver to severe cirrhosis, liver failure and HCC (68). The involvement of NLRP3 inflammasome in ALD has been demonstrated by a robust expression of NLRP3, caspase-1 and IL-1β in alcohol-fed mice, while liver inflammation and steatosis are dramatically attenuated in *NLRP3*^−/−^ or *caspase-1*^−/−^ mice (18). The proinflammatory cytokine IL-1β leads to the recruitment of invariant natural killer T (iNKT) cells, which promotes the influx of neutrophils for exacerbated hepatitis (19).

Beyond regulating IL-1β activation, NLRP3 inflammasome also facilitates the occurrence of pyroptosis (20). It has been confirmed that the pyroptosis determinant protein GSDMD is activated in the liver of mice suffering ALD (69). Adenoviral expression of cleaved GSDMD in hepatocytes aggravates the severity of liver inflammation and damage. Interestingly, one type of miRNA, miR-148a, has recently been demonstrated to suppress pyroptosis in ALD (70). The hepatocyte specific expression of miR-148a by lentivirus delivery directly inhibits the interaction between thioredoxin-interacting protein and NLRP3, leading to attenuated pyroptosis and decreased incidence of ALD (70). These reports provide evidence that NLRP3 inflammasome is pathogenic in the development of ALD, however, the trigger that initiates the activation of NLRP3 remains to be determined.

### Nonalcoholic steatohepatitis (NASH)

NASH is a progressive type of NAFLD with chronic hepatic damage and inflammation (71). The hepatocellular damage is associated with toxic effects induced by accumulated lipids, such as saturated fatty acid (ceramide and palmitate) and cholesterol crystals (72). Emerging evidence has accumulated that NLRP3 inflammasome is activated by these toxic lipids as a pathogenic mechanism for the development of NASH in murine models (21, 73–76). Increased levels of NLRP3, GSDMD and IL-1β are observed in the liver of patients with NASH (77). Beyond amplifying inflammatory responses, activation of NLRP3 inflammasome also promotes liver fibrogenesis during NASH, as blockade of NLRP3 improves NASH pathology by simultaneously suppressing liver inflammation and fibrosis (78). The latest research has demonstrated the importance of GSDMD-mediated pyroptosis in the process of NASH, as *GSDMD*^−/−^ mice develop remarkably attenuated steatohepatitis compared to WT mice, further confirming the detrimental role of NLRP3 inflammasome signaling pathway in NASH (77).

One potential negative regulator of NLRP3 inflammasome during NASH is autophagy (79). Defective autophagy causes the accumulation of dysfunctional mitochondria and increased production of ROS, which is required for the activation of NLRP3 inflammasome (80, 81). In contrast, induction of autophagy by ezetimibe dampens NLRP3 inflammasome activity and ameliorates hepatic lipid accumulation and inflammation on MCD (82). All these findings imply the beneficial role of autophagy in NASH via the suppression of NLRP3 inflammasome.

On the contrary to the above studies suggesting that NLRP3 promotes NASH, an experimental research has shown that NLRP3 deficiency leads to increased bacteremia and aggravated NASH (15). These diverse functions of NLRP3 have been explained by the evidence of different activities of NLRP3 inflammasome in different organs during NASH (83). In the liver, expression of NLRP3 inflammasome is upregulated and responsible for the pathogenesis of NASH, but downregulated in the gut that protects against alteration of intestinal bacteria (83, 84). These observations suggest that liver-specific blockade of NLRP3 inflammasome is necessary to afford improvement in liver inflammation and steatosis but devoid of gut microbial dysbiosis.

### Viral hepatitis (hepatitis B and C)

Hepatitis viruses preferentially infect hepatocytes and cause liver inflammation with high mortality, among which approximately 90% are attributable to chronic infection induced by hepatitis B virus (HBV) and HCV (85). Several studies suggest that NLRP3 inflammasome is the central player in the pathophysiology of viral hepatitis (22, 86, 87). The hepatic expression of NLRP3, caspase-1 and IL-1β are significantly higher in patients with active untreated chronic HBV than those in chronic remission (86). In addition, a strong correlation is demonstrated between levels of IL-1β and severity of liver inflammation in HBV patients, implying that NLRP3-mediated IL-1β is the potential driving force of HBV-induced viral hepatitis.

NLRP3 inflammasome is also identified in HCV infection by the observation of an upregulated expression of NLRP3 signaling pathway in monocytes and macrophages with HCV (87). During HCV infection, NLRP3 inflammasome can serve as either a beneficial or a detrimental role. On one hand, kupffer cell has been identified as the primary cell source of IL-1β in chronic HCV patients, and the production of IL-1β by kupffer cell is associated with amplified inflammatory responses (52). In addition, NLRP3 inflammasome stimulates lipid droplet formation in hepatocytes, which promotes the morphogenesis and replication of HCV, thus contributing to the pathogenesis of liver diseases (22). On the other hand, NLRP3-mediated activation of IL-18 in monocytes stimulates the production of IFN-γ to prime resistance to HCV infection (14). These observations suggest that NLRP3 inflammasome derived from macrophages, hepatocytes and monocytes exerts different functions, which requires in-depth investigations for the potential link of NLRP3 inflammasome activation between these cells in HCV infection.

### Nanoparticle-induced liver injury

The optimal physicochemical properties of nanoparticles make them widely used for disease diagnosis, imaging, and treatment, but their clinical applications are greatly hampered by fulminant hepatitis and liver injury (88). Tremendous efforts are being made to clarify the underling mechanisms, and recent studies have identified that NLRP3-mediated pyroptosis serves as a critical pathogenic factor in the liver injury induced by multiple nanoparticles, including rare-earth oxide (REO), quantum dots and mesoporous silica (23–26). For example, REO treatment activates NLRP3 inflammasome, leading to the occurrence of pyroptosis and secretion of IL-1β in kupffer cells, both of which are suppressed by GSDMD knockdown (24). Coating REO with a small peptide RE-1 inhibits NLRP3 activation via reducing ROS generation and calcium influx, thus attenuating REO-elicited inflammation, further confirming the pathogenic role of NLRP3 in REO-induced toxicity (23). Detailed insights into the mechanism have shown that the sustained activation of NLRP3 in response to REO is mediated by lysosomal damage and massive release of cathepsin B (24). Collectively, these studies implicate the involvement of NLRP3 inflammasome-mediated pyroptosis in nanoparticle-induced liver injury, which may provide novel strategies for controlling nanoparticles-mediated adverse effects.

### Liver fibrosis

Liver fibrosis is a pathogenic result of chronic liver diseases, such as ASH and NASH, and characterized by deposition of extracellular matrix (ECM) (89). Hepatic stellate cells (HSCs) are the primary cells for the storage of ECM, and multiple functional changes of HSCs can be caused by NLRP3 inflammasome, including suppression of chemotaxis, upregulation of collagen and transforming growth factor-β (90, 91). These functions are confirmed by the study that knocking in NLRP3 induces the activation of HSCs and subsequent accumulation of ECM proteins, while fibrogenesis is not reversed by IL-1Ra therapy, indicating that some other regulators of NLRP3 inflammasome pathway instead of IL-1β promote fibrogenesis (20).

In addition to the direct effect on innate immunity, NLRP3 inflammasome has an essential role in shaping adaptive immune responses in liver fibrosis. In particular, IL-1β promotes the differentiation of Th17 cells to secret interleukin-17 (IL-17), which is a critical proinflammatory cytokine in amplifying inflammation responses and perpetuating liver fibrosis driven by NLRP3 inflammasome activation (92). Collectively, NLRP3 inflammasome pathway appears to be central to the pathogenesis of liver fibrosis, and this uncovered link may open avenues for novel therapeutics for liver fibrosis.

## Conclusion

**Figure 1:**
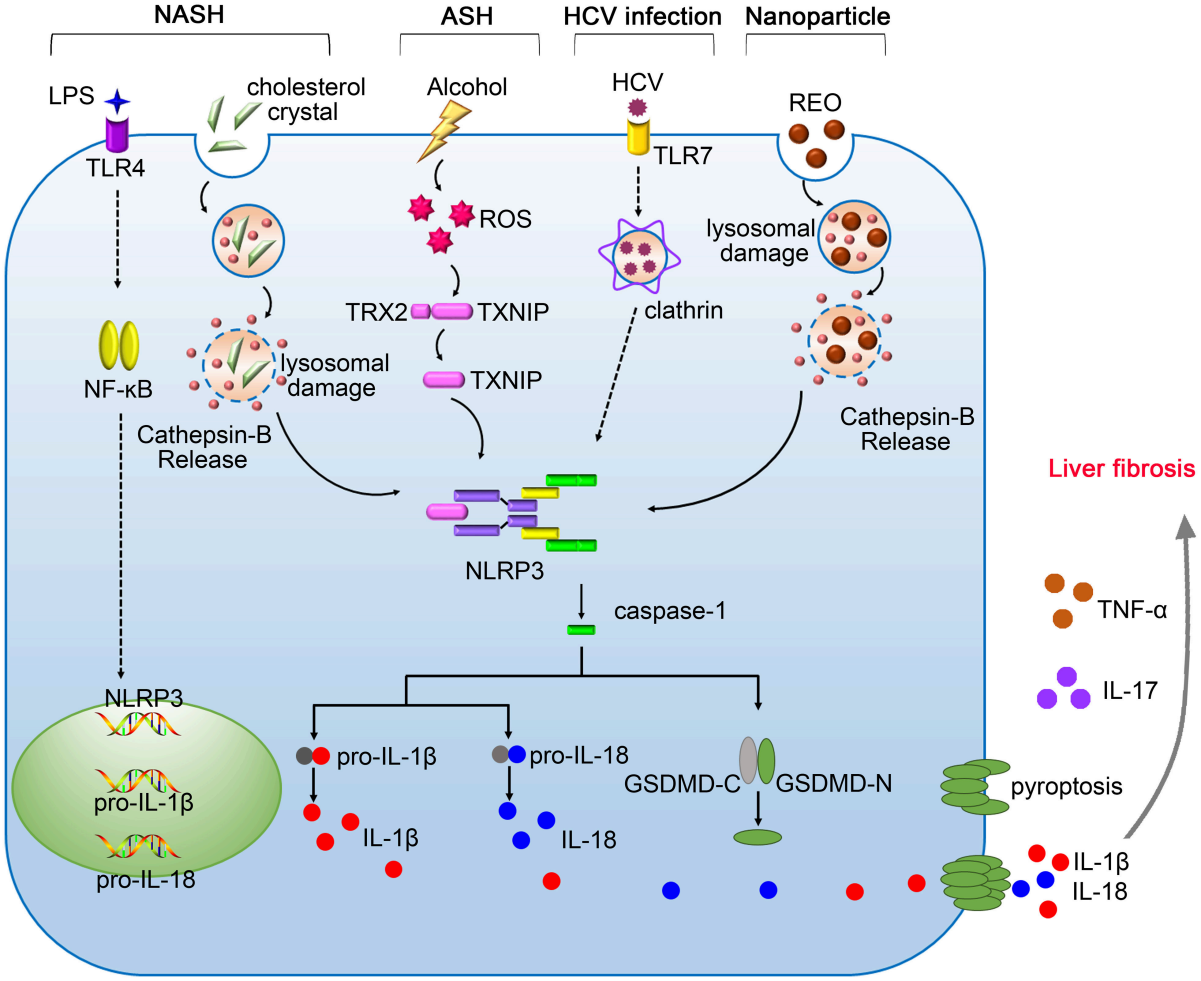
The pathogenic roles of NLRP3 inflammasome in liver diseases. Gut-derived PAMPs, such as lipopolysaccharide (LPS), activate nuclear factor kappa B (NF-κB) signaling pathway, promoting the expression of pro-IL-1β, and pro-IL-18. The NLRP3 inflammasome in the liver is activated by serious danger signals, such as cholesterol crystals, ethanol, and REO nanoparticles. Excessive alcohol consumption stimulates the generation of ROS, which facilitates the cleavage of TXNIP and contributes to assembly of NLRP3 inflammasome. The activation of NLRP3 inflammasome in response to HCV infection requires the recognition by Toll-like receptor-7 (TLR-7) and clathrin-mediated endocytosis. Pyroptosis features GSDMD pores on the membrane, allowing the release of IL-1β and IL-18 into the extracellular space. NLRP3 inflammasome cooperates with TNF-α and IL-17 contributing to the pathogenesis of liver fibrosis.

In summary, inflammasomes are central components of innate immune system that protect liver from pathogen infection, metabolism syndrome, oxidative stress and tumor growth, however, excessive immune responses mediated by inflammasomes may promote the pathogenesis of various liver diseases. The dual functions of inflammasomes pose a challenge in designing inflammasome-based therapies. Therefore, it is important to better understand the precise mechanisms underlying the activation of inflammasomes. To date, the pathological role of NLRP3 inflammasome in liver diseases have been extensively studied (Figure 1). Future investigations need to elucidate the hepatic importance of other inflammasomes, which may hopefully provide new targets for the treatment of liver diseases.

## Author contributions

JL and DJ conceived the review. JL wrote the manuscript. DJ revised the manuscript. All authors have read and approved the final version of the manuscript.

### Conflict of interest statement

The authors declare that the research was conducted in the absence of any commercial or financial relationships that could be construed as a potential conflict of interest.
